# Spectral Photon-Counting Computed Tomography: Technical Principles and Applications in the Assessment of Cardiovascular Diseases

**DOI:** 10.3390/jcm13082359

**Published:** 2024-04-18

**Authors:** Antonella Meloni, Erica Maffei, Alberto Clemente, Carmelo De Gori, Mariaelena Occhipinti, Vicenzo Positano, Sergio Berti, Ludovico La Grutta, Luca Saba, Riccardo Cau, Eduardo Bossone, Cesare Mantini, Carlo Cavaliere, Bruna Punzo, Simona Celi, Filippo Cademartiri

**Affiliations:** 1 Bioengineering Unit, Fondazione G. Monasterio CNR-Regione Toscana, 56124 Pisa, Italy; antonella.meloni@ftgm.it (A.M.); positano@ftgm.it (V.P.); 2 Department of Radiology, Fondazione G. Monasterio CNR-Regione Toscana, 56124 Pisa, Italy; clemente@ftgm.it (A.C.); carmelo.degori@ftgm.it (C.D.G.); mocchipinti@ftgm.it (M.O.); 3 Department of Radiology, Istituto di Ricovero e Cura a Carattere Scientifico SYNLAB SDN, 80131 Naples, Italy; ericamaffei@gmail.com (E.M.); carlo.cavaliere@synlab.it (C.C.); bruna.punzo@synlab.it (B.P.); 4 Diagnostic and Interventional Cardiology Department, Fondazione G. Monasterio CNR-Regione Toscana, 54100 Massa, Italy; sergio.berti@ftgm.it; 5 Department of Radiology, University Hospital “P. Giaccone”, 90127 Palermo, Italy; lagruttaludovico@gmail.com; 6 Department of Radiology, University Hospital of Cagliari, 09042 Monserrato (CA), Italy; lucasabamd@gmail.com (L.S.); riccardocau00@gmail.com (R.C.); 7 Department of Cardiology, Ospedale Cardarelli, 80131 Naples, Italy; ebossone@hotmail.com; 8 Department of Radiology, “G. D’Annunzio” University, 66100 Chieti, Italy; cesare.mantini@gmail.com; 9 BioCardioLab, Fondazione G. Monasterio CNR-Regione Toscana, 54100 Massa, Italy; simona.celi@ftgm.it

**Keywords:** spectral computed tomography, photon-counting detectors, myocardial disease, coronary imaging

## Abstract

Spectral Photon-Counting Computed Tomography (SPCCT) represents a groundbreaking advancement in X-ray imaging technology. The core innovation of SPCCT lies in its photon-counting detectors, which can count the exact number of incoming x-ray photons and individually measure their energy. The first part of this review summarizes the key elements of SPCCT technology, such as energy binning, energy weighting, and material decomposition. Its energy-discriminating ability represents the key to the increase in the contrast between different tissues, the elimination of the electronic noise, and the correction of beam-hardening artifacts. Material decomposition provides valuable insights into specific elements’ composition, concentration, and distribution. The capability of SPCCT to operate in three or more energy regimes allows for the differentiation of several contrast agents, facilitating quantitative assessments of elements with specific energy thresholds within the diagnostic energy range. The second part of this review provides a brief overview of the applications of SPCCT in the assessment of various cardiovascular disease processes. SPCCT can support the study of myocardial blood perfusion and enable enhanced tissue characterization and the identification of contrast agents, in a manner that was previously unattainable.

## 1. Introduction

Computed tomography (CT) is a powerful diagnostic imaging tool that plays a crucial role in the detection, characterization, and monitoring of various medical conditions [1]. Conventional CT (single-energy) primarily focuses on delivering anatomical and morphological evaluations of tissues and organs, relying on a qualitative assessment of tissues in comparison to the surrounding structures [2]. The acquired images are contingent upon the selected energy level during the acquisition process. Spectral CT goes beyond conventional CT:—it acquires and analyzes data at multiple energy levels or wavelengths, enabling the differentiation of materials based on their unique energy-dependent attenuation characteristics [3,4]. The extraction of energy-dependent spectral information is called material decomposition and it provides detailed information about the composition of tissues, allowing for improved tissue characterization and enhancing diagnostic capabilities in several ways [5]. Moreover, spectral CT enables the accurate assessment of iodine concentration within tissues and the subtraction of iodine-specific information from contrast-enhanced images, allowing for a clearer visualization of anatomical structures and a better quality of anatomical images using mono-energetic reconstructions [4,6]. Mono-energetic reconstructions contribute to improved image sharpness and reduced artifacts, resulting in clearer and more diagnostically valuable representations [6,7]. The heightened level of detail offered using spectral CT facilitates a more nuanced understanding of anatomical structures and pathological conditions, thereby enhancing diagnostic accuracy and potentially opening new avenues for tailored therapeutic interventions.

Dual-energy computed tomography (DECT), which involves acquiring two datasets with different energy levels (typically high and low kVp), represents the first successful implementation of spectral CT. All the different available DECT platforms employ X-ray detectors that integrate the energy of incoming X-rays (energy-integrating detectors—EIDs) and, therefore, necessitate the integration of supplementary components or techniques, including additional beams, detectors, or filters, to achieve spectral separation [2,8,9,10].

Recently, the introduction of spectral photon-counting CT (SPCCT) has opened up new possibilities for spectral imaging. The core innovation of SPCCT lies in its photon-counting detectors (PCDs), which provide a unique methodology for acquiring and processing X-ray data. By counting individual photons and measuring their energy directly, PCDs surpass the capabilities of traditional energy-integrating detectors.

The objective of this narrative review, prepared in accordance with the indications provided in [11], is to summarize the basic technical principles and the benefits associated with SPCCT technology and to provide a brief but comprehensive overview of the applications of SPCCT in the assessment of different cardiovascular diseases.

## 2. Spectral Photon-Counting CT Technology

### 2.1. Technical Aspects

**Figure 1:**
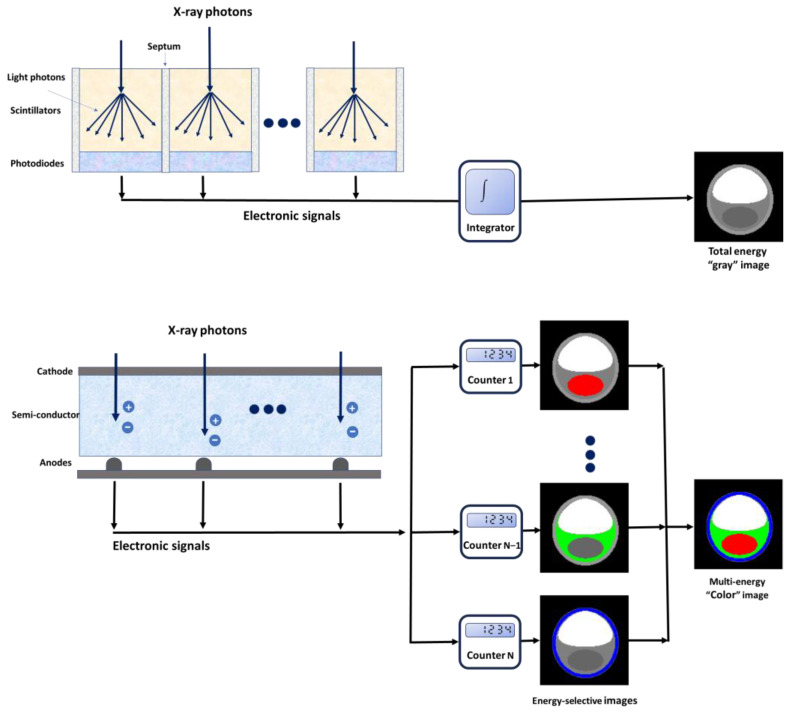
Schematic representation of a photon-counting detector, which directly converts X-rays into an electrical signal (**top**) and a conventional energy-integrating detector (**bottom**).

PCDs are constructed using semi-conductive materials, such as cadmium telluride, cadmium zinc telluride, or silicon [12], which possess the unique capability to directly convert incoming photons into electrical charges [13,14,15] (Figure 1). The application of an external voltage, typically ranging between 800 and 1000 V, between the cathode (located on the upper side) and the pixelated anode electrodes (situated on the lower side) creates a potent electric field within the detector. When an X-ray photon interacts with the detector material, it generates a charge cloud comprising electron-hole pairs. The charge clouds are propelled towards the pixelated anode electrodes by the electric field and induce short current pulses. An electronic pulse-shaping circuit is employed to amplify and transform the induced current pulses into voltage pulses [16]. The shaped electrical pulses have heights directly proportional to the energy of the absorbed X-rays. A “counter” mechanism is employed to quantify the pulses that exceed a predetermined energy threshold. PCDs employ multiple energy thresholds and perform a comparative analysis of all pulses, categorizing incident photons into distinct energy groups or bins [17]. This process enables the differentiation of X-ray photons based on their energy levels. In contemporary PCCT systems, the energy bins or channels typically range from 2 to 8. The choice of the number of energy bins can depend on the specific design and application of the PCCT system; the optimal number may vary for different imaging scenarios and clinical objectives.

EIDs are made of scintillator elements that convert X-rays into visible light [15,18,19]. This emitted light is subsequently detected by a photodiode array constructed from a semi-conducting material. This array produces an electrical signal that is directly proportional to the total deposited energy, which also includes electronic thermal noise. The electrical signal undergoes amplification and is subsequently converted into a digital signal, allowing for further processing in tomographic image reconstruction. It is important to note that, due to the integration of energy from all incident photons within a specific time interval, the detector loses any information regarding the energy of individual X-ray photons. Optically opaque partitions called septa are integrated between scintillating detector pixels, with the primary objective of preventing light cross-talk between these neighboring elements [19].

### 2.2. Benefits Associated with PCDs

The energy-resolving capabilities of PCDs offer several advantages over conventional EIDs, including the elimination of electronic noise, the improvement of contrast between different tissues, the correction of artifacts caused by beam hardening, and the possibility to perform multi-energy/multi-parametric imaging.

#### 2.2.1. Improved Image Quality (Noise, Contrast, and Artifacts)

Different from EIDs, PCDs typically have an energy threshold, which is the minimum energy level required for an X-ray photon to be registered by the detector. The threshold is set to be approximately 20–25 keV, which is notably higher than the background noise level of the electronic system associated with the PCD. As a result, electronic noise does not significantly influence the count rates of the PCD [17]. The noise mitigation capabilities of PCDs contribute significantly to improved image quality, making them particularly valuable in situations requiring minimal radiation exposure and when imaging patients with larger body sizes, where image clarity and diagnostic accuracy can be challenging, due to increased attenuation [20,21].

PCDs apply uniform weighting to the counted X-ray quanta. Uniform weighting implies that all X-ray photons meeting the minimum energy requirement are treated equally regarding their contribution to the detector signal [16,22]. On the contrary, EIDs use linear weighting and low-energy photons make a diminished contribution to the overall detector signal. Since the information pertaining to the distinction between various materials is primarily focused within the lower range of X-ray energy, the PCD-CT technology presents an enhanced contrast and a superior contrast-to-noise ratio (CNR) when compared to the conventional EID-CT. This superiority is particularly evident when imaging materials characterized by low X-ray attenuation, such as iodine [12,23,24,25]. The contrast discrepancy related to iodine between PCD-CT and EID-CT becomes more conspicuous at higher X-ray tube voltages. Importantly, in PCDs, the customization of the weighting scheme, which consists of assigning distinct weights to photons of varying energy levels, allows the optimization of the CNR for a specific material [16,26,27].

Beam-hardening artifacts represent one of the most common artifacts, caused by the increased attenuation of low-energy photons compared to high-energy photons [28]. This dissimilarity in attenuation results in distortions within the reconstructed CT images, manifested as streaking or shading artifacts [29]. PCDs effectively mitigate the impact of beam hardening through constant weighting, which allows the normalization of attenuation measurements across diverse energy levels [30,31]. In particular, PCDs demonstrate optimal immunity to beam-hardening effects when employing high-energy thresholds [13,20,32].

The remarkable improvement in imaging quality positions PCCT as a promising technique for pioneering new low-dose imaging protocols. The reduction in radiation dose is a critical factor in CT, as the potential risks associated with patient exposure to radiation must be carefully weighed against the necessity for high-quality diagnostic images. Indeed, several studies have proved the ability of PCCT to effectively retain a high level of image quality, while simultaneously reducing radiation dose [21,33,34,35].

#### 2.2.2. Multi-Energy Acquisition

Material decomposition is one of the main mechanisms used in spectral CT. Material decomposition algorithms break down unknown tissues into specific materials, based on their unique X-ray attenuation characteristics at various energy levels. The number of materials or bases corresponds to the amount of spectral information that has been collected (N bases for N spectral data) and it can be augmented to N + 1 by applying mass or volume conservation restrictions [36], which may potentially introduce inaccuracies in the material decomposition process [37]. Material decomposition algorithms generate material-specific images, which can display and measure the presence of particular elements in a CT volume, and energy-selective images. Preselected materials (e.g., iodine, fat, and calcium) can be subtracted from material-specific images. The removal of the iodine leads to the generation of virtual non-contrast (VNC) images, which exhibit an image quality comparable to that of true non-contrast images [38,39] and potentially eliminate the need for a dedicated unenhanced acquisition, decreasing both radiation dose and scan time. On the other hand, iodine can be superimposed onto grayscale images with a color gradient, generating iodine maps that specifically highlight the distribution and concentration of iodine within the scanned area [40,41]. Energy-selective applications include virtual monochromatic images (VMIs), which emulate scans acquired at a single energy, described in terms of kiloelectronvolt [42,43,44]. Low-keV images enhance iodine contrast and lesion conspicuity, albeit with the trade-off of increased noise. Conversely, high-keV images exhibit less contrast but mitigate beam-hardening artifacts [6,45]. Images captured at intermediate energy (60–75 keV) offer balanced contrast and image noise and are ideal for the assessment of soft tissues [46].

By collecting data in two energy regimes, EIDs have the capability to distinguish up to three types of materials within the imaged area [36]. In addition, DECT is susceptible to spectral overlap, a factor that diminishes the accuracy of material decomposition. Conversely, PCDs, thanks to their ability to differentiate photons with different energies through pulse height analysis, have the potential to discriminate ≥3 materials accurately and to enable multi-energy spectral CT without spectral overlap [47]. Increasing the number of energy regimens in spectral CT leads to superior material-specific or weighted pictures, enabling a more accurate identification of the underlying tissue composition and unlocking new possibilities for diagnostic imaging [48,49].

The other important benefit associated with the use of multiple energy measurements in spectral CT is the possibility of adding additional materials to the spectral decomposition of the images based on their distinct K-edge energies. This approach, called K-edge imaging, relies on adjusting the acquisition energy thresholds to capture the target materials’ unique energy shifts at the K-edge [50]. K-edge imaging is a real breakthrough in CT imaging, providing opportunities for the use of non-iodine contrast agents, such as gold, silver, platinum, bismuth, and ytterbium [51,52,53,54], and for the development of new types of contrast agents, such as nanoparticles targeted to particular cells or enzymes [55,56,57,58]. These opportunities open a completely new CT approach for simultaneous multi-contrast agent imaging and functional and molecular imaging [59,60,61,62]. Multi-material imaging enables the determination of the precise distribution of different contrast agents administered simultaneously or to picture various contrast agents with distinct distribution properties at one time [19,22]. Molecular imaging provides detailed insights into the dynamic interactions and activities occurring within cells and molecules in real time, providing the in vivo characterization and measurement of biological processes not achievable with “classical” diagnostic imaging [55,56,57,58,63].

#### 2.2.3. Enhanced Spatial Resolution

In addition to their spectral imaging capacities, PCDs provide enhanced spatial resolution compared to EIDs. Indeed, in EIDs, the need to mitigate the impact of signal loss arising from the “dead zones” represented by the septa, which cannot be made excessively thin, imposes constraints on the smallest achievable size of the active detector elements. In PCDs, the elimination of non-responsive areas between pixels enables a reduction in pixel size, without compromising geometric efficiency [16]. The pixel pitch can be refined to values as small as 0.15–0.225 mm at the isocenter [64,65,66,67].

### 2.3. Current Challenges of PCCT

#### 2.3.1. Technical Challenges

Current technical challenges of PCDs include cross-talk effects (k-escape, charge-sharing, or fluorescence) and pulse pile-up, which can affect the accurate determination of the energy of the incident photons [18,68].

Charge sharing occurs when the charge cloud arrives close to the border between two pixels and is captured and registered by multiple adjacent pixel electrodes [50,69]. This unintended sharing of the signal can result in a misallocation of the signal, as it may be mistakenly attributed to multiple pixels rather than being precisely assigned to a single pixel, introducing inaccuracies in determining the location and energy of the incident radiation [70]. Compton scattering and fluorescence involve the emission of secondary photons, after the interaction of an incident photon with the detector. Rather than being confined to a single pixel, these secondary photons tend to be detected in neighboring pixels, giving rise to situations where multiple events jointly share the overall energy of the incident photon [17]. The likelihood of K-fluorescence and Compton scattering is influenced by the material composition of the detectors: K-fluorescence is more accentuated in detectors characterized by higher atomic numbers, such as cadmium telluride, and Compton scattering is more accentuated in detectors made of silicon [19]. These effects introduce various challenges, encompassing inaccuracies in energy assignment to individual pixels, potential undercounts or overcounts, compromised spatial resolution due to the dispersion of counts among adjacent pixels, and associations between energy bins in different pixels [50]. Charge sharing and cross-talk from secondary photons become more likely as pixel size diminishes and, consequently, limit the attainable minimum size of pixels in PCDs.

Pulse pile-up effects manifest when numerous photons interact with the detector in a very short time span, preventing the detector from distinguishing individual events. This leads to the incorrect registration of multiple low-energy charges as a single high-energy photon [71,72]. The errors introduced in photon counting and energy measurement have the potential to distort energy spectra and compromise image quality. Mitigating pulse pile-up can be achieved by decreasing the pixel size of the detector, consequently decreasing the number of incident photons per detector channel. Anyway, the influence of pulse pile-up is generally negligible under the usual X-ray flux rates encountered in clinical CT imaging [73].

#### 2.3.2. Alternative to Iodine-Based Contrast Agents

Some clinical applications of PCCT require the use of an alternative to iodine-based contrast agents [55,58]. Alternative contrast agents offer advantages such as improved tissue differentiation, better visualization of specific pathologies, or reduced risks for patients with contraindications to the administration of iodinated contrast media.

Gadolinium-based contrast agents, used in magnetic resonance imaging (MRI), can also be used for diagnostic CT angiography (CTA), as an alternative to iodinated contrast materials [74]. However, there are some limitations to using gadolinium in CTA, including the lower vascular contrast attenuation compared to conventional CTA using an iodinated contrast, which can make it more challenging to visualize certain details, and the requirement of high doses of gadolinium [74,75]. Compared to MRI, CT requires a significantly increased dosage of gadolinium to achieve adequate contrast enhancement and this could have implications for safety and cost considerations.

Heavy elements like gold and bismuth have been employed as contrast agents in preclinical and experimental settings [53,55,76,77], but their safety profiles for human use are still under investigation and regulatory scrutiny.

## 3. Cardiovascular Applications

### 3.1. Coronary Artery Assessment

Coronary CT has emerged as the primary diagnostic tool for investigating coronary pathologies, recommended as the first-line examination [78]. However, persistent challenges exist in accurately assessing the coronary lumen in the presence of heavily calcified plaques or small stents and in analyzing plaque components.

The presence of severe calcification can lead to blooming artifacts in traditional imaging, potentially rendering examinations inconclusive or resulting in an overestimation of stenosis [79]. In this scenario, the PCCT’s ability to mitigate blooming artifacts has been proved particularly beneficial. In the phantom study by Koons et al., replicating coronary arteries containing calcifications of varied sizes and shapes, PCCT offered improved visualization of calcium plaques and a clearer depiction of the patent lumen compared with conventional CT at an equivalent dose [80]. Additionally, in the case of a ring-shaped plaque inducing a substantial 75% reduction in the vessel’s cross-sectional area, only images generated using PCCT could discern the presence of iodine within the lumen, underscoring the distinctive capability of PCCT in detecting partial blockages that might go unnoticed using conventional CT. Si-Mohamed et al. confirmed, in their in vivo study (14 patients), that the mitigation of blooming artifacts achieved using PCCT resulted in an improved estimation of lumen permeability [81]. Li et al. presented an innovative methodology employing the material decomposition of multiple-energy CT images to quantify the percentage of stenosis [82]. The phantom experiments demonstrated that the four-threshold PCCT approach was more effective in reducing estimation errors than DECT and two-threshold PCCT, especially in the presence of dense calcifications. Furthermore, employing a three basis material decomposition model on the four-threshold PCCT images led to the creation of distinct maps illustrating the distribution of calcium, iodine, and water, enhancing the specificity of material identification and providing valuable insights into the composition of the imaged structures.

**Figure 2:**
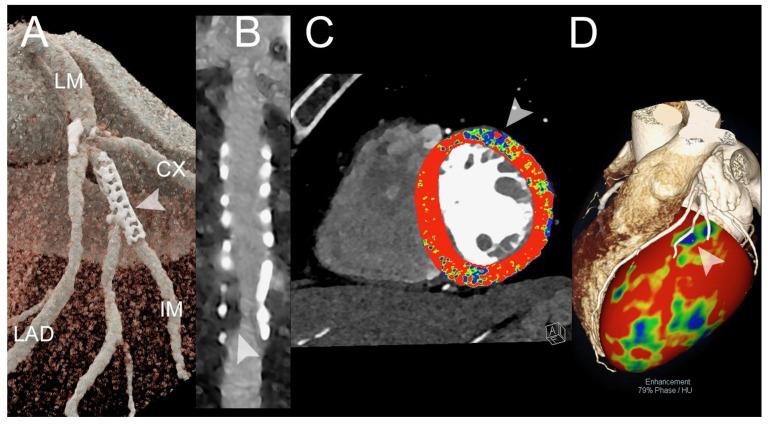
Spectral cardiac CT angiography of coronary arteries and left ventricle in a patient with previous PCI. The figure shows a spectral CT angiography in a patient with chronic pulmonary embolism displayed with 3D-cinematic rendering (**A**), a stretched MPR along the central lumen line of the intermediate branch (IM) with a proximal stent (arrowheads in (**A**)) (**B**), the first pass rest perfusion image (spectral monochromatic+ 40 KeV with color-coded perfusion overlay) (**C**), and) the overlay of 3D-volume rendering with the anatomy of the coronary arteries and color-coded perfusion map (**D**). The figure shows a significant in-stent restenosis (arrowhead in (**B**)) that causes segmental perfusion delay/defect in rest first-pass perfusion (arrowhead in (**C**)). The correspondence between the restenosis and the perfusion defect is displayed in (**D**) (arrowhead). The scan was performed on a commercial whole-body Dual Source Photon-Counting CT scanner (NAEOTOM Alpha, Siemens Healthineers, Erlangen, Germany), with 0.2/0.4 mm slice thickness, 0.1/0.2 mm reconstruction increment, FOV 140–160 mm, resolution matrix of 512 × 512/1024 × 1024 pixels on the source axial reconstructions with a kernel filtering of Bv48-60-72 (vascular kernel medium-sharp) and with maximum intensity of Quantum Iterative Reconstruction (QIR 4); the scan is performed with retrospective ECG gating with tube current modulation. The displayed spatial resolution is 0.10/0.20 mm. Abbreviations: PCCT = Photon-Counting CT; PCI = percutaneous coronary intervention; ECG = electrocardiographic.

Although CT is a useful non-invasive imaging technique for patient follow-up after coronary artery stent implantation, the presence of blooming artifacts arising from stent struts, attributed to partial volume averaging and beam hardening, poses challenges in accurately evaluating the stent lumen and identifying in-stent patency and in-stent restenosis (ISR) [83]. The in vitro comparative study by Mannil et al. demonstrated that, when compared to traditional CT using the same imaging parameters, PCCT acquisitions offered several benefits, such as improved stent lumen clarity, lower noise, less blooming artifacts, and a better overall image quality [84]. Moreover, a superior visualization of the coronary stent lumen and a decrease in metal blooming artifacts have been demonstrated for UHR PCCT, in comparison to standard-resolution PCCT and conventional EID-CT by several independent studies [85,86,87,88,89]. Feuerlein et al. examined a complex scenario in which a PCCT system with six energy thresholds was used to scan a phantom that resembled a low-density calcified plaque inside a coronary metal stent and showed attenuation similar to that of the gadolinium-filled vascular lumen [90]. Gadolinium K-edge imaging made it possible to distinguish between intravascular gadolinium-based contrast agent, calcified plaque, and stent material, as well as to successfully reduce beam-hardening artifacts. The advantages of PCCT in terms of reduction in blooming artifacts and accurate visualization of both the stent and the coronary lumen have also been confirmed in vivo [81,91,92]. The in vitro study of Bratke et al. demonstrated the benefits of PCCT for the non-invasive detection of ISR [93]. Both conventional CT and PCCT were able to identify the soft-plaque-like stenoses inserted into the coronary stents embedded in a contrast-filled vessel phantom. However, with PCCT, it was possible to accurately define the residual lumen for seven stents, something that was never possible with traditional CT. The enhanced capabilities of PCCT should lead to a reduction in the need for invasive coronary angiography, especially in cases involving heavily calcified coronary arteries and small stents. Figure 2 shows a PCCT example of significant in-stent restenosis causing a perfusion deficit.

The coronary artery calcium (CAC) score serves as a robust indicator of atherosclerotic burden and as a consistent and reproducible means of assessing risk for major cardiovascular outcomes [94,95]. The Agatston method [96], calcium volume determination [97], and calcium mass score determination [97] are the three primary approaches for quantifying the CAC score, with the Agatston score being the most commonly utilized. PCDs have shown promise in improving the accuracy of CAC score quantification, which, in conventional CT scanners, is affected by partial volume effects that impair the identification of thin calcifications, leading to an underestimation of the CAC burden, and blooming artifacts that cause an overestimation of the CAC burden. First, phantom studies demonstrated the PCCT’s enhanced capabilities in the detection and characterization of small or subtle calcifications, compared to conventional CT systems [98,99]. Of note, it has been shown that monoenergetic reconstructions on PCCT can accurately evaluate the CAC and that there is a decline in the CAC score when QIR strength increases and monoenergetic levels rise [100]. In an ex vivo study using cadaveric hearts, Agatston scores from PCCT and conventional CT were found to have a strong correlation and agreement, as well as a good inter-scan reproducibility [101]. Another comparative ex vivo study showed the capability of UHR PCCT to reduce calcium blooming artifacts and to increase the accuracy of CAC quantification, significantly reducing the mean absolute percent error of volume estimations, in comparison to traditional CT [102]. Importantly, PCCT has been proved able to successfully maintain a high degree of sensitivity in CAC detection, while concurrently lowering radiation dose [103,104,105,106]. The reduction in the risks associated with the ionizing radiation exposure may open the way for the implementation of the CAC screening in asymptomatic individuals for the detection of occult coronary artery disease.

Since PCCT has inherent spectrum capabilities, it can reconstruct images without the iodinated contrast signal, eliminating the necessity for an unenhanced scan and, thus, reducing the patient’s overall radiation dose burden. Emrich et al. evaluated the precision in CAC scoring of a new virtual non-iodine (VNI) reconstruction technique called PureCalcium, in comparison with virtual non-contrast (VNC) reconstructions and true non-contrast (TNC) acquisitions [107]. A good agreement between the TNC and PureCalcium approaches was demonstrated in the phantom setting; the in vivo investigation with 67 patients showed that the use of PureCalcium reconstructions, as opposed to VNC reconstructions, significantly increased the accuracy of CAC score categorization and the precision of CAC quantification. According to a recent phantom study assessing the impact of cardiac motion, Agatston scores derived from VNI were less affected by the presence of cardiac motion, and VNI reconstructions consistently exhibited superior performance compared to VNC reconstructions for all heart rates, leading to a lower underestimation of CAC scores compared to the actual calcium mass [108].

Although CAC scoring is a surrogate marker of the atherosclerotic plaque burden, it is not useful in identifying a vulnerable plaque [109]. The vulnerable or high-risk plaques are those plaques more susceptible to rupture leading to vascular thrombosis, which is considered to be the principal mechanism that accounts for acute myocardial infarctions or sudden coronary deaths [110]. Unstable plaques differ from stable lesions and are associated with a large necrotic core, a thin fibrous cap (less than 65 μm), inflammation (prevalently in the form of macrophage infiltration), angiogenesis, plaque hemorrhage, microcalcification, and positive remodeling determining luminal stenosis of <75% [111]. It has, therefore, been suggested that taking into account both plaque burden and type may improve atherosclerosis imaging and risk prediction [112,113,114,115]. Broadly, imaging assessments of plaque type can be divided into anatomic evaluations of plaque composition and molecular data regarding disease activity.

Conventional EID-based CT, coupled with sophisticated segmentation software, can help distinguish various components of coronary artery plaques based on their X-ray attenuation properties. This may include identifying features such as calcified plaques, non-calcified plaques, and mixed plaques [116,117]. However, using standard CT imaging, non-calcified plaque components, such as fibrous and fatty tissue, often exhibit substantial overlap in their density profiles, making it challenging to differentiate them accurately, and the partial volume effect makes quantitative measurements challenging [118]. Moreover, coronary plaques are usually composed of three to four materials and current algorithms cannot decompose a mixture of four materials. SPCCT can address these challenges. The performances of PCCT and traditional EID-CT were compared in an in vitro study by Rotzinger et al., where three distinct patient sizes (small, medium, and large) were simulated [119]. Thanks to its lower noise and increased spatial resolution, PCCT allowed for the improved recognition of simulated non-calcified and lipid-rich coronary plaques in all scenarios. In the study by Bouseel et al., 10 calcified and 13 lipid-rich non-calcified plaques from post-mortem human coronary arteries were scanned using PCCT [120]. Through the analysis of the dissimilarities in spectral attenuation and the iodine-based contrast agent concentration, PCCT allowed for the clear identification of normal arterial walls, lipid-rich plaques, calcified regions, and adjacent adipose tissue. The in vivo study of Mergen et al. confirmed the accuracy of coronary plaque extraction from SPCCT images [121]. In particular, the use of the ultra-high-resolution mode enabled a significant reduction in blooming artifacts, with a consequent enhanced visualization of non-calcified plaque constituents [121]. In a study involving a cohort of 51 patients, Vattay and colleagues investigated the impact of VMIs derived from PCCT on attenuation values and the corresponding volumes of plaque components [122]. In comparison with polychromatic images at 120 kVp used as a reference, a higher degree of similarity was observed when employing higher VMI levels (100–180 keV) for non-calcified plaque volumes, 70 keV for calcified plaque volumes, and low-energy images (40–50 keV) for lipid-rich atheromatous plaque volumes.

Disease activity (inflammation and neovascularization) in coronary plaque can be assessed using molecular imaging techniques. Preliminary phantom and animal studies have demonstrated the potential of SPCCT, using K-edge imaging in combination with gold nanoparticles, to provide important physiological data at the molecular and cellular levels. The study by Cormode et al. was the first to demonstrate, on a variety of phantoms and apo E-KO mice models of atherosclerosis, the ability of PCCT to differentiate in an accurate way among a gold nanoparticle contrast agent specific for macrophages, an iodine-based contrast agent, and calcified tissue [56]. The study conducted by Si-Mohamed and colleagues on atherosclerotic and control New Zealand white rabbits, scanned both before and two days after administering gold nanoparticles, revealed an enhanced correlation between gold concentration and macrophages when employing PCCT compared to traditional CT acquisitions (0.82 vs. 0.41) [64]. Moreover, the differentiation between the enhancement of the inner lumen with an iodinated contrast agent and the enhancement of the vessel wall with gold nanoparticles, validated through transmission electron microscopy and inductively coupled plasma optical emission spectrometry, was achieved solely using PCCT utilizing gold K-edge imaging.

### 3.2. Myocardial Perfusion

One of the most interesting capabilities of spectral CT is the use of the different absorption characteristics of the iodinated contrast at different kV levels to create a myocardial iodine map. Since myocardial iodine concentration has the potential to be a useful surrogate marker for myocardial perfusion, the quantification of the myocardial blood pool can be performed based on the amount of iodine per voxel [123]. Iodine distribution is commonly displayed using a color-coded map, which reflects the iodine distribution across all myocardial segments, according to the American Heart Association (AHA) model. Subsequently, the relative normalized myocardial attenuation density and the TPR (Tissue Perfusion Ratio) can be derived from these maps. DECT myocardial perfusion studies conducted under rest and stress protocols have demonstrated good accuracy compared to diverse reference modalities [124,125,126,127].

**Figure 3:**
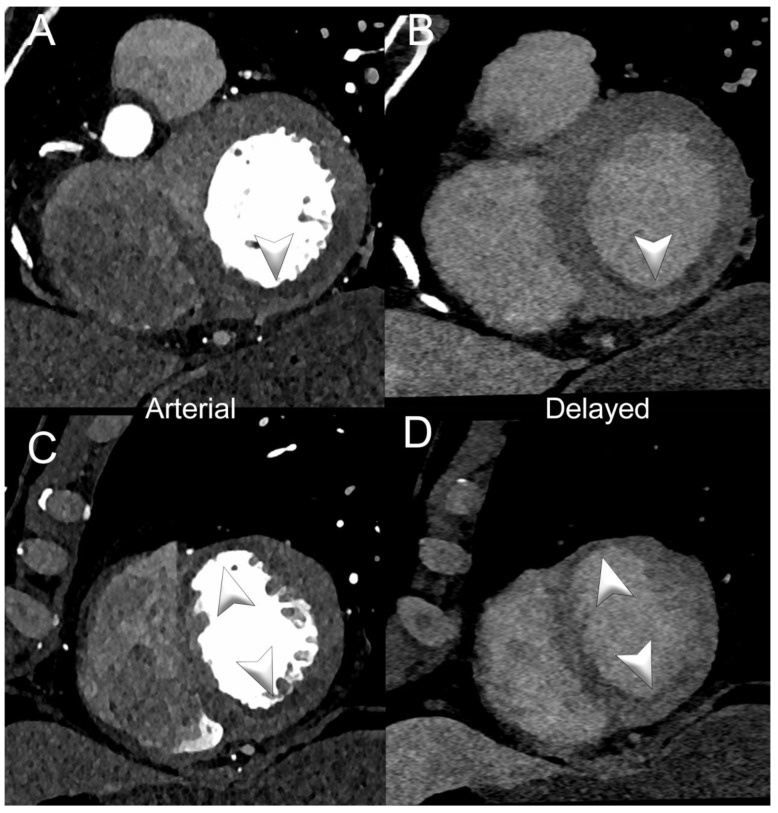
Spectral cardiac/coronary PCCT example of sub-acute LV ischemia. The figure shows a patient with a recent acute myocardial infarction in the territory of the right coronary artery and of the left anterior descending coronary artery. Panels (**A**,**B**) show a short-axis view of the base of the left ventricle (LV) in arterial/angiographic phase (**A**) and delayed phase (**B**); panels (**C**,**D**) show the same information in the apical section of the LV. Arrowheads indicate the multiple LV segments with hypoperfusion (early and late). The delayed phase was performed with spectral acquisition and monochromatic+ 40 KeV reconstruction. The scan was performed on a commercial whole-body Dual Source Photon-Counting CT scanner (NAEOTOM Alpha, Siemens Healthineers), with 0.2/0.4 mm slice thickness, 0.1/0.2 mm reconstruction increment, FOV 140–160 mm, resolution matrix of 512 × 512/1024 × 1024 pixels on the source axial reconstructions with a kernel filtering of Bv48-60 (vascular kernel medium-sharp) and with maximum intensity of Quantum Iterative Reconstruction (QIR 4); the scan is performed with retrospective ECG gating with tube current modulation. The displayed spatial resolution is 0.1/0.20 mm. Abbreviations: PCCT = Photon-Counting CT; LV = left ventricle; ECG = electrocardiographic.

**Figure 4:**
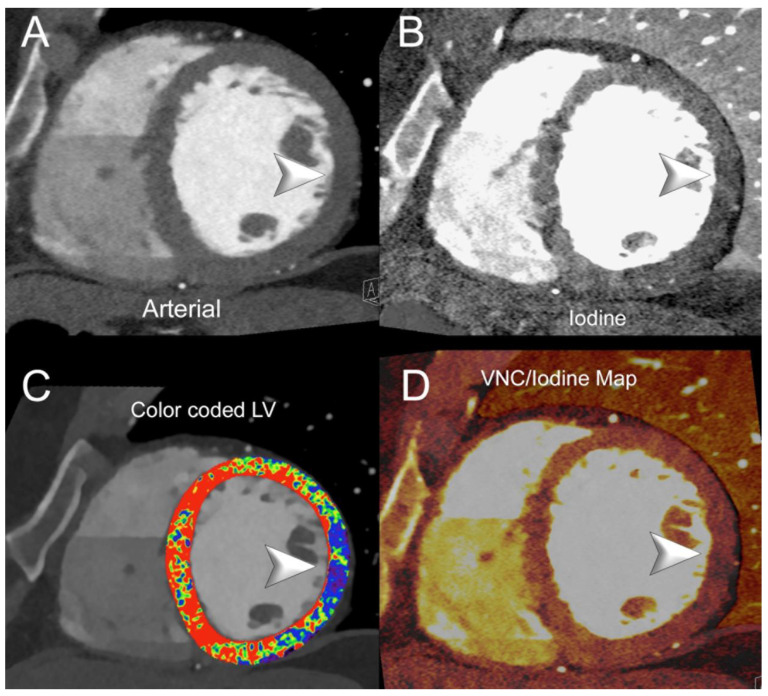
Spectral cardiac/coronary PCCT example of chronic LV ischemia. The figure shows a patient who underwent a cardiac CT for the assessment of coronary artery disease, displaying a delayed perfusion at rest in the territory of the circumflex coronary artery (lateral wall of the LV). Panel (**A**) shows a mid-basal short-axis view of the left ventricle (LV) in arterial/angiographic phase; panel (**B**) shows the same projection with iodine distribution; panel (**C**) shows the standard image with color-coded LV map; and panel (**D**) shows the same projection with mixed virtual non-contrast/iodine map overlay. Arrowheads indicate, in each figure, the sharp perfusion delay (early) in the lateral wall of the LV. The delayed phase was performed with acquisition and Spectral Monochromatic+ 40 KeV reconstruction. The scan was performed on a commercial whole-body Dual Source Photon-Counting CT scanner (NAEOTOM Alpha, Siemens Healthineers), with 0.2/0.4 mm slice thickness, 0.1/0.2 mm reconstruction increment, FOV 140–160 mm, resolution matrix of 512 × 512/1024 × 1024 pixels on the source axial reconstructions with a kernel filtering of Bv48-60 (vascular kernel medium-sharp) and with maximum intensity of Quantum Iterative Reconstruction (QIR 4); the scan is performed with retrospective ECG gating with tube current modulation. The displayed spatial resolution is 0.1/0.20 mm. Abbreviations: PCCT = Photon-Counting CT; LV = left ventricle; ECG = electrocardiographic.

Figure 3 and Figure 4 show SPCCT examples of left ventricular ischemia.

A phantom study demonstrated a good accuracy of PCCT, in terms of iodine quantification in iodine maps and of generation of CT numbers in virtual monochromatic images [43].

In a recent case report involving a 61-year-old male presenting with acute chest symptoms, dual-energy-derived iodine maps from PCCT showed a diminished iodine concentration in the midventricular inferolateral wall [128]. This observation suggested an insufficient blood supply to that region and was confirmed using CMR, which depicted a small ischemic transmural scar in the same area.

### 3.3. Myocardial Tissue Characterization

Changes in tissue composition are a hallmark of various diseases affecting the myocardium. These alterations include the development of myocardial fibrosis, the existence of edema, or the infiltration of iron, fat, or amyloid into the heart.

There are two primary forms of myocardial fibrosis—interstitial fibrosis and replacement fibrosis.

The interstitial (diffuse) fibrosis involves the abnormal accumulation of collagen and other extracellular matrix components in the interstitial space, due to non-ischemic or infiltrative cardiomyopathies [129]. The quantification of myocardial extracellular volume (ECV) provides a non-invasive means to measure the proportion of extracellular matrix present in the whole myocardium and is, therefore, employed as a surrogate marker of interstitial fibrosis. Cardiac magnetic resonance (CMR) is widely recognized as the “gold standard” for non-invasive ECV assessment, but ECV quantification with CT has recently emerged as a robust and reliable alternative [130,131]. Compared to CMR, the main advantages of CT are the lower cost, the wider availability, the fast scanning speed, the possibility to generate volumetric 3-dimensional image data with high resolution and isotropic voxels, and the increased image quality and readability in patients with metal implants or devices [132]. The ECV quantification relies on determining the ratio between the concentration of the contrast medium (gadolinium in CMR and iodine in CT) in the myocardium and the plasma portion of the blood pool. In conventional EIDs, two distinct methods are currently used to quantify ECV. The single-energy or subtraction approach requires the acquisition of pre-contrast or non-enhanced images and late enhancement (LE) images, obtained 3 to 5 min after the contrast administration [133]. The differences in Hounsfeld units (∆HU) between the pre- and the post-contrast acquisitions are used to assess the distribution of the contrast medium by using the formula ECV = (1 − Hematocrit) * (∆HUmyocardium/∆HUbloodpool) [134]. Validation of this approach against both CMR and histopathology has been performed [135,136]. In dual-energy CT, the spectral separation of LE scans acquired simultaneously with two different kV and mA settings allows for material decomposition. The iodine distribution equilibrium in the myocardium and blood pool after contrast can be directly quantified on iodine maps obtained from the late enhancement scans [137]. The ECV calculation formula used for spectral imaging acquisition is: ECV = (1 − Hematocrit) * (Iodinemyocardium/Iodinebloodpool). This method obviates the need for a non-enhanced acquisition and is free of issues connected to misregistration during the manual tracing of the regions of interest [138]. A good agreement has been demonstrated between ECV quantified with DECT and CMR, although DECT tends to a slight overestimation [139,140]. The study by Mergen et al. was the first to demonstrate, in vivo, the feasibility of ECV quantification with PCCT at a low radiation dose [141]. In a cohort of 30 patients with severe aortic stenosis, the single-energy- and the dual-energy-derived ECV measurements showed a strong correlation (r = 0.87, *p* < 0.001), with a small mean difference of 0.9% and narrow limits of agreement. The average body mass index of the study population was 28 ± 5 kg/m^2^ and the median radiation dose for the LE scan was 1.2 mSv. This radiation dose is considerably lower than that one reported in recent studies where the ECV quantification was performed using an EID-CT system—4.7 mSv [142] or 5.2 mSv [138]. The study by Aquino et al., involving 29 patients scanned with PCCT, confirmed the strong association between dual- and single-energy-based ECV measurements (r = 0.91, *p* < 0.001) and demonstrated that the dual-energy approach offers the potential to reduce radiation exposure by 40% [143]. Importantly, compared to CMR, both the subtraction and spectral methods showed a robust correlation and a high reliability for ECV quantification.

**Figure 5:**
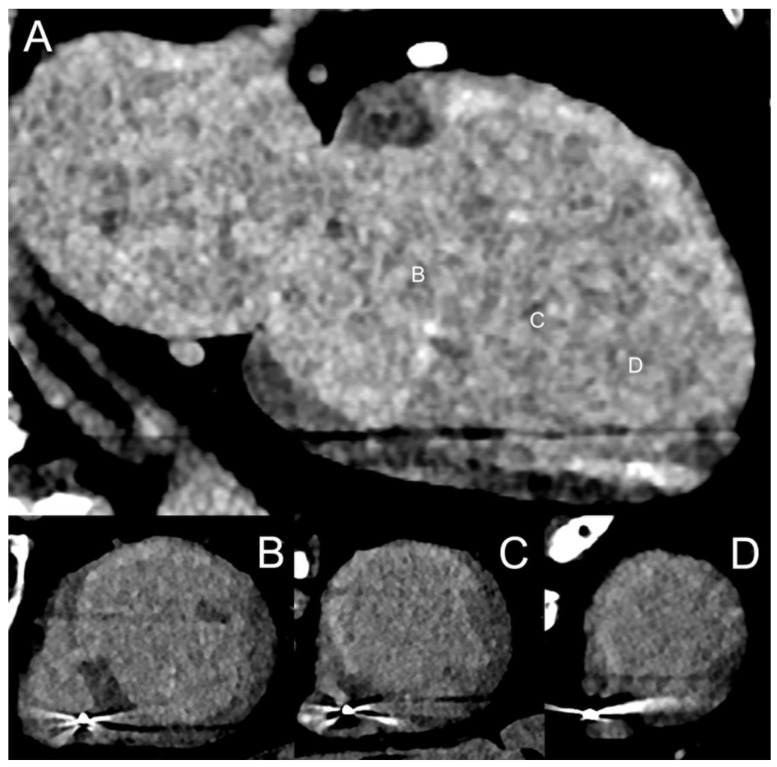
Spectral cardiac/coronary PCCT example of chronic post-infarction cardiomyopathy of the LV. The figure shows a patient who underwent a cardiac CT to assess the status of coronary artery disease and the viability of the left ventricle (LV; **A–D**). Panel (**A**) shows a 2-chamber long-axis view of the left chambers of the heart with extensive near transmural ischemic delayed enhancement of the anterior wall of the LV in the delayed phase; panels (**B**–**D**) show the short-axis view of the LV at the basal, mid, and apical sections of the LV. The delayed phase was performed with spectral acquisition and monochromatic+ 40 KeV reconstruction; in this case, the patient also has an intracardiac device; however, there are no issues in terms of safety or image quality. The scan was performed on a commercial whole-body Dual Source Photon-Counting CT scanner (NAEOTOM Alpha, Siemens Healthineers), with 0.2/0.4 mm slice thickness, 0.1/0.2 mm reconstruction increment, FOV 140–160 mm, resolution matrix of 512 × 512/1024 × 1024 pixels on the source axial reconstructions with a kernel filtering of Bv48-60 (vascular kernel medium-sharp) and with maximum intensity of Quantum Iterative Reconstruction (QIR 4); the scan is performed with retrospective ECG gating with tube current modulation. The displayed spatial resolution is 0.1/0.20 mm. Abbreviations: PCCT = Photon-Counting CT; LV = left ventricle; ECG = electrocardiographic.

Replacement (scar) fibrosis usually develops in the later stages of the disease following irreversible myocyte death or damage. Myocardial delayed enhancement CT has shown promise in the evaluation of myocardial scar tissue [144]. Figure 5 shows an SPCCT example of chronic post-infarction cardiomyopathy of the left ventricle. However, late gadolinium enhancement CMR remains the reference standard modality for this purpose, thanks to the increased CNR and the ability to clearly differentiate between healthy myocardium and scar tissue [145]. In a canine model of myocardial infarction, Symons et al. showed that PCCT could distinguish between the two administered contrast agents (iodine and gadolinium-based) with great clarity [60]. The integration of gadolinium, iodine, and soft tissue maps enabled the authors to achieve an excellent contrast between infarcted myocardium, remote myocardium, and LV blood pool. The comparison of PCCT results with histology and CMR confirmed that the scar tissue was accurately delineated.

### 3.4. Epicardial and Pericoronary Fat

Epicardial adipose tissue (EAT) is the fat deposit between the myocardium and the visceral layer of the pericardium. Pericoronary adipose tissue (PCAT) specifically refers to the adipose tissue surrounding the coronary arteries. These fat depots are metabolically active and have been linked to coronary artery disease and the development of high-risk plaque [146,147,148,149,150]. Moreover, the PCAT has emerged as a marker of vascular inflammation with significant prognostic value [151]. The changes in adipose tissue composition can be evaluated from standard CT images using different approaches, including quantifying the thickness, volume, and attenuation of the adipose tissue [152].

The in vivo study conducted by Risch et al. showed that the use of VNC reconstructions derived from PCCT datasets provided a reliable method for assessing EAT volume, exhibiting a strong correlation and only minor differences when compared to traditional TNC reconstructions and offering the additional advantage of a significant decrease in the patient’s radiation dose [153]. Moreover, the calcium-preserving algorithm developed in the study demonstrated better and more uniform outcomes in estimating EAT volume when compared with the conventional algorithm.

Mergen and his team investigated the influence of monoenergetic energy levels on PCAT measurements in a cohort of 30 patients scanned with a first-generation whole-body PCCT system [154]. They found that, as the energy level increased, there was an increase in the PCAT mass attenuation surrounding the right coronary artery, left anterior descending artery, and circumflex artery, as well as a reduction in the mean attenuation difference between unenhanced and contrast-enhanced scans.

### 3.5. Radiomics

Radiomics is a quantitative approach to medical imaging, operating under the assumption that biomedical images contain intricate information regarding disease-specific mechanisms, which are imperceptible to human eyes and cannot be accessed through conventional visual examination. It involves the high-throughput extraction and analysis of a vast array of quantitative features, including textural information [155]. Since extracting reliable and insightful texture features relies on requisites such as optimal spatial resolution and SNR [156], PCCT offers the potential to improve radiomic analyses based on CT imaging data.

Ayx et al. compared PCCT and conventional CT of radiomics features extracted from the human heart [157]. A good similarity was found in first-order radiomics features depicting fundamental statistical properties of image intensity. In contrast, higher-order radiomics features, which capture more intricate patterns and relationships within the images, exhibited notable differences. In another in vivo study including 30 patients, the same group examined the myocardial changes linked to CAC score using a PCCT dataset [158]. The differentiation between patients with and without coronary artery calcification was achievable using four distinct radiomics-based textural characteristics. Furthermore, the direct relationship between the increase in complexity of the texture feature and the increase in Agatston score suggested that the myocardial texture becomes more heterogeneous and exhibits a higher variance in its structural patterns, as the calcification burden increases.

In the study conducted by Dunning et al., 19 patients underwent CCTA, using PCCT and VMIs at 50 keV, 70 keV, and 100 keV, and the reconstruction of VNC images and iodine maps was performed [159]. A comprehensive set of 93 radiomic features was extracted from each image and was subsequently compared between low- and high-risk plaques identified by an expert radiologist. The VMIs at 100 keV and the VNC images exhibited the highest accuracy in classifying coronary plaque risk, due to the reduced presence of iodine and calcium in these images.

## 4. Conclusions

Spectral photon-counting computed tomography holds tremendous potential for revolutionizing cardiovascular imaging and improving patient care and outcomes, by providing detailed and high-quality diagnostic information and offering unprecedented opportunities for the identification, characterization, and staging of cardiovascular diseases.

Although the number of publications describing the clinical applications of SPCCT is rapidly growing, they remain limited. Moreover, the existing studies primarily featured small sample sizes and did not perform a longitudinal follow-up analysis assessing the added value of PCCT compared to conventional CT in terms of prognosis and risk stratification. The wider adoption and the conduct of large cross-sectional and prospective clinical studies represent the key to fully realizing the potential of this transformative technology in clinical practice.

## Data Availability

Not applicable.
